# Exploring Factors Associated With the Stalled Implementation of a Ground-Up Electronic Health Record System in South Africa: Qualitative Insights From the E-Tick Case Study Using the Consolidated Framework for Implementation Research (CFIR)

**DOI:** 10.2196/73831

**Published:** 2026-01-12

**Authors:** Campion Zharima, Frances Griffiths, Jane Goudge

**Affiliations:** 1 Centre for Health Policy, Faculty of Health Sciences, University of the Witwatersrand, Johannesburg, 2193, South Africa; 2 Centre for Primary Health Care Studies, Warwick Medical School, University of Warwick, Medical School Building, CV4 7AL, Coventry, United Kingdom, 44 2476522534

**Keywords:** electronic health records, implementation, barriers, facilitators, Consolidated Framework for Implementation Research, CFIR

## Abstract

**Background:**

Electronic health records (EHRs) have the potential to improve service delivery through record keeping and monitoring health outcomes. As countries move toward universal health coverage, digital health tools such as EHRs are essential for achieving this goal. However, EHR implementation in middle-income countries like South Africa faces obstacles.

**Objective:**

This study explores the reasons behind a stalled implementation of the electronic tick register (E-tick) system (an electronic version of a paper primary health care register to record services provided), using the Consolidated Framework for Implementation Research.

**Methods:**

Using a qualitative design, in-depth interviews were conducted with 38 participants to explore their perceptions and experiences, and the factors surrounding the success and stalling of E-ticks. Participants included managers, stakeholders, implementers, and end users from the 3 implementation clinics. Data was collected using semistructured interview guides. The Thematic and Consolidated Framework for Implementation Research framework analysis (innovation, inner setting, individual characteristics, implementation process, and outer setting) was applied.

**Results:**

The E-tick system was designed to improve data quality in paper health registers, addressing inaccuracies in reporting to district and provincial health departments (Innovation domain). Implementers iteratively developed the system through user input from managers and clinicians, and stakeholder engagement of software developers, funders, health managers, and decision-makers from the provincial health department (individual characteristics). Although the system was initially well adopted by end users, it stalled primarily due to outer setting factors, which included a change of developers, funding cuts, and limited support at the provincial health department level due to capacity gaps, political appointments, and mistrust stemming from corruption and abuse of the tender system. Moreover, resistance to leveraging lessons from locally developed small-scale systems further constrained institutional support for the E-tick.

**Conclusions:**

Although successful implementation of EHRs can be facilitated by strong user engagement and co-design, outer setting factors such as governance, funding, and policy alignment can pose significant threats to sustainability. This underscores the importance of effective synergy between top-down and bottom-up processes for successful implementation.

## Introduction

### Overview

Electronic health records (EHRs) hold the promise of improving service delivery, monitoring and evaluation of health interventions, and record-keeping for public health [1]. As more countries work towards universal health coverage, leveraging digital health tools like EHRs becomes more important for enhancing service delivery and improving quality of care [2,3]. The World Health Organization’s global digital health strategy emphasizes the need for accelerated adoption of EHRs to achieve health goals [4]. However, EHR implementation in many middle-income countries remains slow due to financial and technological challenges [5].

Implementing EHRs is an evolving process that requires adaptive and context-specific policies to meet ever-changing needs [6], and a clear vision to move from pilot to scaled implementation [2]. In some high-income countries where implementation has occurred [7], a “top-down” approach has typically been used, where systems are preconfigured, and users must adapt. Although this offers coordination and central oversight, it can limit flexibility. In contrast, bottom-up approaches emphasize co-design with end users and adaptation to local workflows, which often enhances usability and adoption [8]. Research suggests that successful EHR implementation in complex health systems requires balancing national coordination with local innovation, as this allows centralized oversight, while engaging local health communities and tailoring systems to user needs [8,9].

South Africa has made policy advances, such as the National Digital Health Strategy (NDHS) (2019‐2024) [10], with considerable resources as a middle-income country and sufficient technical expertise, but progress toward a national EHR has been slow. This is because policy direction and technical capacity are sometimes impeded by systemic and political barriers. Within this context, most primary health care facilities continue to rely on manual paper-based systems, which are labor-intensive and prone to errors in data collection [11,12]. However, bottom-up efforts have been made to improve data quality and accuracy. These include Tier.net, an electronic record system used with patients living with HIV and tuberculosis, and the electronic tick register (E-tick), the electronic record system we studied. The E-tick is a digital version of the tick register, a book in each health care facility for recording patient attendance and care delivered. In this paper, we use the Consolidated Framework for Implementation Research (CFIR) to explore an attempted implementation of the E-tick, a system conceptually similar to an EHR, examining how its implementation stalled despite a consultative approach in its implementation.

### Background

#### Digital Health Policy in South Africa

As a middle-income country, South Africa has made policy strides in line with the global developments, which should facilitate the adoption of EHRs, such as the National Health Act 61 of 2003 and the NDHS (2019‐2024) [10,13]. The Health Act outlines the role of the National Department of Health in coordinating health information systems across national, provincial, and district levels [10]. Although policy direction is set at the national level, the federal system means that provinces can independently determine their own health care spending. Therefore, implementation largely occurs at provincial and district levels, with each maintaining its own health information systems within the national framework. This provincial autonomy has contributed to fragmentation, as different provinces and regions develop EHR systems through separate procurement processes [14]. This fragmentation is further compounded by a political economy of procurement, in which tenders are prone to corruption, mismanagement, and the absence of oversight structures [15]. These factors directly affect which innovations are funded, how resources are distributed, and whether systems can successfully reach scale, as funding decisions often prioritize large-scale centralized systems.

South Africa uses 2 health information systems in the public sector: the Health Patient Registration System (HPRS) and the District Health Information Software (DHIS; developed through a collaboration between the University of the Western Cape and the University of Oslo), jointly developed by the University of the Western Cape and the University of Oslo [16]. The HPRS is a manual patient registry that records patient contact details as well as legal identification numbers, while the DHIS is an electronic system used to collect aggregated data on services provided to patients in public health facilities [17]. The DHIS data is first collected manually, then captured into the DHIS software, where it is submitted to the district, provincial, and national health departments. The method used to record primary health care data is a tick register, a manual paper-based system where clinicians mark or tick off the services provided in a large logbook with columns for dates and types of services offered (antenatal care, vaccinations, or chronic disease management). However, this process can be arduous, time-consuming, and prone to many recording errors. Studies evaluating the DHIS have confirmed persistent data gaps, citing poor documentation, manual entry under heavy workloads, and errors as major contributors to discrepancies [18].

#### South African Health System

South Africa plans to implement a National Health Insurance fund to facilitate better purchasing decisions through more formal contracting relationships rather than the historical allocation of budgets by provinces [19]. In this case, EHRs are key in monitoring patient outcomes and assessing the value of care purchased. Although most provinces have had some type of operational EHR system in public hospitals [20], there is no national EHR at the primary care level, and different architectures still pose a challenge to their integration for nationally coordinated use [21].

Provincial health departments in South Africa are often run by political appointees, who work under directives from their affiliated political party. This has led to pervasive corruption in the country’s health system, which has been reported at various levels of government [22]. For example, 150 million rand awarded to a communications company during the COVID pandemic was misappropriated, and the health minister was suspended as a result [23]. In another case, the provincial chief financial officer who exposed a 1-billion-rand tender fraud was assassinated [24]. Such cases illustrate how poor financial management systems and political appointments enable corruption, which acts as an impediment to the public trust in the health system [25]. For digital health innovations, such dynamics increase the risk that systems are chosen for political or financial interests rather than their impact or merit, which affects the health department’s ability to work with other stakeholders, or source funds for large-scale projects, including projects to implement EHRs. Moreover, the absence of countermeasures such as accountability and transparency makes it difficult to implement large-scale projects like EHRs.

#### The E-Tick System

An electronic version of the paper-based tick register (E-tick) was developed and piloted in 3 public health care facilities in the Ekurhuleni district between 2017 and 2021. Clinicians record services provided directly into the digital system on a patient’s record, which also allows facility managers to monitor daily activities such as headcount and services offered. Although this is not a full EHR, the system can be expanded to include diagnosis and lab results and to integrate with other systems that are currently used in public health facilities. Against this backdrop, the E-tick system sought to provide an alternative that could improve data accuracy at the facility level while feeding directly into the existing provincial and national reporting systems. However, such progress is dependent on not only user adoption factors but also broader contextual dynamics [26]. Thus, by situating the E-tick in relation to both micro-level realities and macro-level policy and political economy, we aim to provide a more holistic understanding of why grassroots digital health interventions may stall or fail.

### Consolidated Framework for Implementation Research (CFIR)

Implementation research seeks to understand why some implementation efforts succeed and others do not, and the role of the context [27]. To explore the key factors affecting implementation in our case study, we used the CFIR, which integrates existing theories of implementation and behavior change into a unified framework [28]. The CFIR is a meta-theoretical tool with standardized constructs to examine enablers and barriers to implementation across different stages (preimplementation, during implementation, and postimplementation) [29].

The CFIR was developed through a review of published theories and reports on factors influencing implementation [30]. Hence, its strength lies in its broad applicability and potential for generalizability, which are useful for informing practice across diverse settings. The framework has 5 broad domains (Table 1), which include: innovation domain, inner setting domain, outer setting domain, individuals domain, and the implementation process domain, which together capture multilevel determinants of implementation [31]. Due to the framework’s flexibility, researchers can tailor it to specific interventions and contexts [32], and it has been widely adopted in research, applied across different research designs, and cited in over 300 published articles [29]. Recent updates have been made to the framework, using responses from authors who used the framework, leading to refined construct names and definitions to make them more encompassing [31]. In this paper, we draw on the updated CFIR to guide both our analysis and reporting of findings.

**Table 1.** Consolidated Framework for Implementation Research (CFIR) domains by Damschroder et al [28].

CFIR domains	Definition
Innovation	The “thing” being implemented, such as a new treatment, program, or service. Key factors that affect innovation include its source, evidence base, advantages over existing practices, adaptability, trialability, complexity, design, and cost.
Individuals	The roles and characteristics of people involved in innovation. Key roles include high-level leaders, mid-level leaders, opinion leaders, facilitators, and implementation teams who guide and support the innovation. It also highlights innovation deliverers and recipients.
Inner setting	The environment where an innovation is implemented, such as a hospital or school. Key elements include structural characteristics like physical and IT infrastructure, work organization, and staffing levels.
Outer setting	The broader context or setting within which an organization or project operates, such as a hospital system or school district. Factors influencing implementation in the Outer Setting include large-scale disruptions, local attitudes, socioeconomic conditions, external partnerships, and regulatory policies.
Implementation process	The activities and strategies to introduce and sustain an innovation. Key components include forming teams to collaborate on tasks, assessing the needs of both innovation deliverers and recipients, and evaluating the context to identify barriers and facilitators. Planning, tailoring strategies, engaging participants, testing small-scale changes, and reflecting on outcomes are critical steps.

## Methods

This study adopted a qualitative design to explore the perceptions and experiences of external stakeholders, senior health managers, implementers, and end users of the E-tick system.

### Participants and Sampling

We recruited a total of 38 participants, categorized into three groups: (1) managers and stakeholders, (2) implementation team, and (3) end users. The senior health managers and external stakeholders category included decision makers and higher-level people who were not directly involved in the system’s implementation but provided insights into the context and the decisions impacting it. The implementation team comprised individuals who were responsible for initiating and deploying the system and introducing it to the users, while the end user team consisted of the clinicians and managers from 3 recipient facilities. Participants from the managers and stakeholders, as well as the implementation team, were purposively selected through referrals from key individuals and recruited via email. For end users, convenience sampling was used, with the researcher recruiting available users with the aid of gatekeepers during facility visits. Our sample intentionally included stakeholders from multiple levels of the health system to minimize elite bias, ensuring that the perspectives of senior health managers were balanced with those of frontline users and mid-level implementers. Below is a summary table of the participants in this study (Table 2).

**Table 2.** Study participants (N=38).

Category and roles	Count (n)
Senior managers and stakeholders	
National, provincial and district health managers	7
Parastatal staff	2
NGO^a^ managers and staff	4
Academics	2
Private sector managers	2
Implementers	
Originators	2
IT Technician	1
Data capturers	3
End users	
Facility managers	3
Administrative officer	1
Nurses and midwives	9
Admin clerks	2

a NGO: Non-Governmental Organization.

### Data Collection and Management

Data collection took place between November 2021 and June 2022, which was after the E-tick project had stalled. Thus, participants, particularly implementers and end users, reflected retrospectively on the system’s development, piloting, and discontinuation. Due to COVID-19 health restrictions at the time, some of the interviews with senior health managers and the implementation team were conducted via web using video conferencing software. Interviews with end users were conducted in person in health care facilities during work hours and were conducted primarily in English, which is the standard working language in the facilities. However, participants were free to use local language terms or expressions, which were translated during transcription to preserve meaning.

Different semistructured interview guides were used for the 3 categories of participants. For the managers, we inquired about the broader digital health policy landscape, existing health information systems, government readiness, and feasibility for EHR implementation and the necessary next steps to advance EHR adoption in South Africa. For the implementation team and end users, the interview guides covered topics on the purpose and origins of the E-tick, its piloting rollout, experiences with implementation and daily use, training and support, perceived benefits and challenges, and overall recommendations for improvement. Interviews varied in length, ranging from 10 to 64 minutes. Shorter interviews (under 20 min) were mostly with busy frontline staff, including clerks and clinicians during clinic hours, who provided concise but relevant insights into system use. Longer interviews were with senior health managers with authority over policy alignment, resource allocation, and procurement processes, and implementers who facilitated implementation in the 3 health facilities. Despite these differences, data saturation was reached across stakeholder groups, as no new themes emerged in later interviews. All interviews were audio recorded, and recordings were stored in a password-protected computer to ensure privacy. Audio recordings were transcribed using transcription software and checked for accuracy, and transcripts were anonymized by removing participant-identifying information.

### Data Analysis

The first author led the coding, followed by regular debriefing meetings with coauthors, who are also academic supervisors, to review coding and theme development, which strengthened reliability. In this case, coauthors read all the transcripts, and discrepancies were discussed until consensus was reached. The interview transcripts were coded using NVivo qualitative analysis software (QSR International). Through this process, emergent issues were identified and used to develop the themes, following a 6-step thematic analysis process [33]. An inductive approach guided the thematic analysis, with broad topics derived from the interview guide, leading to the 4 stages of implementation reported in this study (Purpose and origins, Initial development, Piloting and expansion, and Halt).

The accounts from the two originators formed the foundation, providing the background story and backbone of the implementation narrative, whose detailed recollections provided a chronological anchor for mapping implementation processes. These accounts were not taken at face value, but were supplemented by other participants’ accounts, which were used to triangulate and integrate the information into a cohesive story.

After coding, the CFIR was applied to the analysis, linking the framework’s 5 domains to the implementation process as reported in the study’s findings (Table 3) [31]. The CFIR framework guided our analysis and reporting of findings. In this case, CFIR constructs were operationalized as sensitizing concepts to guide coding, with data excerpts being coded against relevant constructs, while leaving space for inductive subthemes to emerge. Thus, the analysis combined deductive coding (using CFIR constructs) with inductive coding to capture novel themes. Multiple coders reviewed transcripts, with regular team discussions to minimize confirmation bias.

**Table 3.** Implementation stages and applied Consolidated Framework for Implementation Research (CFIR) domains.

Stages of implementation	Description	Applied CFIR domains
Origins and purpose of the system (2015‐2017)	Conception of E-tick^a^ by system originators; designed to digitize the existing tick register and reduce errors.	Innovation Individuals Inner setting Outer setting
Initial development (2017‐2019)	Early technical build and adaptation of the system, involving stakeholder input and limited resource mobilization.	Individuals Inner setting Outer setting Implementation process
Piloting (2019‐2020)	Deployment of E-tick system in 3 health facilities.	Individuals Inner setting Implementation process
Halt (2021)	Expansion beyond pilot sites stalled due to barriers to scale up and institutionalization.	Inner setting Outer setting

a E-tick: electronic tick register.

### Ethical Considerations

Ethics approval for this study was granted by the University of the Witwatersrand Medical Human Research Ethics Committee (M210213). Additional permissions were obtained from the Ekurhuleni district research committee and the Gauteng Provincial Department of Health (GP_202106_035). The study was conducted in accordance with local, institutional, and national regulations governing research involving human participants, as well as the principles of the Declaration of Helsinki. All participants provided written informed consent prior to participation. The study purpose, procedures, potential risks, benefits, and participants’ rights, including the right to withdraw at any time without penalty, were explained before consent was obtained. Participant privacy and confidentiality were maintained as interviews were conducted in private settings, and no identifying information was included in transcripts. All data were anonymized, securely stored on password-protected devices, and were only accessible to the research team. Participants did not receive any compensation for their participation.

## Results

### Overview

This section presents the findings, which are ordered according to the domains of the CFIR: innovation, inner setting, individual characteristics, implementation process, and outer setting. This structure allows us to highlight how factors at multiple levels and stages of implementation shaped the processes, outcomes, and ultimately the stalling of the E-tick system. To illustrate the distribution of themes across the framework, Multimedia Appendix 1 provides a summary of implementation stages, CFIR domains and applicable constructs, thematic findings, and illustrative quotes.

### Origins and Purpose of the E-Tick: Innovation and Individuals Domains (2015-2017)

#### Identifying the Problem

The development and implementation of the E-tick was initiated and led by a senior district manager and supported by a senior technician who was employed by the funder, labeled as Originators 1 and 2. The senior manager explained the rationale behind the project:

*Annually, the National Department of Health audits our data for accuracy, and we’ve always had poor audit reports. The biggest problem was the quality of the data. That’s why we started the E-tick, to improve the data quality.*

[Originator 1]

The source of poor data quality was traced back to the tick register: “The data was very poorly filled into the tick register, the services provided not counted properly.” (Originator 1). This was the stage where most errors occurred: “The data issues started in the consultation room, and then the counting and the transferring of the data into the DHIS accounted for about eighty percent of the mistakes.” (Originator 1). Some clinicians echoed these remarks as one shared: “The purpose was to reduce workload and be able to say, reduce patients' waiting time by doing away with pen and paper and to be able to keep our records digitally, which makes it easier to keep them.” (Nurse 12 – AR).

The second originator further describes how decision makers often overlook the importance of effective data collection processes:

*The problem in South Africa is that the people who decide where the funding goes don’t take data management records management seriously. They want this wealth of data so they put in these big paper registers, but they don’t look at the process it takes to get that data*.

[Originator 2]

This includes having too many registers:

*We had twenty-seven different registers, like the antenatal register for a mother and child, and a chronic disease register for HIV. The problem was that while completing all the registers, users were creating their own registers because some information was missing*.

[Originator 1]

Nurses similarly described the burden, with one stating:

*So with the E-tick it was one less writing system… our patients were registered, all the follow up visits are registered, and… it gave a clear history of the patient.*

[Nurse 13 – MM]

#### Finding the Solution

Against this backdrop, the idea of the E-tick began with an invitation to a staff development project to discuss potential solutions to data quality problems:

*We received an invitation because we already recognized a problem with data quality in our tick registers. They proposed several ways to enhance the system, involving different levels of information. While some suggestions were overly complex and resembled an Electronic Health System*.

[Originator 1]

Eventually, the originators decided to make use of an Excel-based E-tick system:

*The organization that developed the District Health Information System …had an Electronic Tick Register and we thought we can use that to improve the data quality. But the system was just an excel sheet*.

[Originator 1]

Thus, the originators built on the Excel-based system:

*We decided to improve the front end, the user interface for the clients. That’s how we developed the E-tick. It was basically to collect the data electronically and to be able to feed it directly into the DHIS, in a way that is easy for the nurses in the consultation rooms*.

[Originator 1]

End users confirmed that the system was simpler and helpful. One nurse explained:

*It was user friendly. If only we would use E-tick only… we will definitely be perfect.*

[Nurse 10 – AR]

Another shared:

*And it was also user friendly. It wasn’t like I’m talking from somebody that’s not very computer literate, basically… it made it very easy, accessible.*

[Nurse 13 – MM]

### Initial Development of E-Tick System: Inner Setting and Individual Domains (2017-2019)

#### Stakeholder Engagement

The first originator approached a nonprofit organization for funding to get the project off the ground: “After our experience, we approached organization X for financial assistance and shared our ideas with them, as [they were already] our long-term funding partner deeply involved in data collection” (Originator 1). After the funders agreed, this was followed by meetings with software developers for planning: “Once the funders agreed, we met with [software] developers to explore system development possibilities, discussed utilizing the HPRS as our registration system.” (Originator 1). It was at this point that the second originator, who worked for the funding organization, became involved. Together, the two originators represented 2 groups of stakeholders, one with the Department of Health and all staff at recipient facilities, and the other with the funding organization and developers:

*[Originator 1] was very good on the clinician side and I was very good on the software side. I had been working with my team [funders] for many years before we started the E-tick, and I developed many systems with them*.

[Originator 2]

As an iterative process, the originators worked closely between clinicians and developers:

*In a normal software development cycle, you scope your business requirements, hand it over to the team, and they will go develop what they think you want and then return to show it to you. We took a different approach. We were involved in the development, sitting with them every step of the way instead of waiting for weeks for changes. This worked extremely well.*

[Originator 2]

Several clinicians further confirmed this engagement, with one manager explaining:

*The first early step was that we had meetings which included facility managers, other stakeholders and our partner [funder], to explain to us what wants to be introduced, which is when we were first given the idea of the E-tick and what it’s supposed to do.*

[Administrative officer 19 – RK]

The originators then selected the facilities for piloting involving managers in the process: “We used random selection and cluster sampling, through a session with all operational clinic managers, who actively participated in this process.” (Originator 1). A manager from one of the facilities described their involvement: “We were called to a district meeting, and this was pitched, the intention was discussed with us... So the facilities that were going to be pilots were selected.” (Assistant Director 17 – RK).

#### System Development

After selecting the facilities for piloting, the system was taken to the recipient facilities as one clinician recalls:

*They called us for like a mini meeting to inform us about E-tick and told us that we were one of the only clinics currently using it, and they were piloting to see if it’s going to work.*

[Nurse 10 – AR]

In the meetings, one of the goals was to find ways to streamline the patient consultation:

*We focused on what made it easy for clinicians to help the patients without them waiting too long. But a bonus that came out of the system is that the system was also able to retrieve a patient’s entire history*.

[Originator 2]

The clinicians provided feedback:

*The clinicians had great ideas. The first time we had the screens ready, we took it back to the clinicians and asked them if this is what they wanted, they approved it and gave suggestions for changes, which we did*.

[Originator 2]

Several engagements were made with the provincial Department of Health to gain support and input on issues such as data storage:

*When we began the pilot, we engaged the Provincial office and kept them informed about the program. A provincial manager suggested storing the data on provincial servers instead. However, they weren’t prepared to proceed at that time*.

[Originator 1]

To ensure this was possible later, the developers ensured the system was compliant:

*We looked at the Departments of Health security and ICT standards, we worked with the Department of Health ICT team as well, and their data team*.

[Originator 2]

However, this did not go as planned, as the data center was not ready in time:

*The idea was to eventually transfer the system and its data to the department’s data center once it was established. However, when we developed it, there was no data center for us to put it in, and we had complied with all the national standards for the developing systems*.

[Originator 2]

### Piloting and Expansion: Inner Setting and Implementation Process Domains (2019-2020)

#### Planning and Configuration

In all 3 chosen facilities, the originators conducted an infrastructural assessment before beginning the pilot:

*We had to check that all computers were working, and that they all had network. The developers didn’t want to have a local server for each clinic because it’s too complicated. So they changed the system to an online system that worked with the cloud*.

[Originator 1]

Some of the existing infrastructure was from the Department of Health:

*We only had to get the computers for the Provincial clinics. So that already made life much easier. Because every clinic, every clinician had a computer, or most of them*.

[Originator 1]

A technician from the health department was involved in configuring them:

*My role was to check infrastructure, to see if it’s there. But they also installed some private infrastructure because of some policies with our network which would require a lot of approvals before we can run it*.

[IT Technician]

This was further supplemented by the funding to improve the infrastructure:

*They [funders] assisted us and made sure that each room had a network cable. Sometimes there were no plugs, so they put in the plugs. They put in the routers*.

[Originator 1]

The planning and configurations also informed decisions on devices that were to be used:

*We wanted to see what the nurses and the clinicians felt more comfortable with, the tablets or the desktops. Due to a lack of space in some consultation rooms, which were too small to put a big desktop, it was easier to mount a tablet onto the wall*.

[Originator 2]

#### Training and Learning

A comprehensive user training was provided in recipient facilities, which was tailored for specific roles:

*There were different training categories, because the roles were different like, clerks, clinicians and so on. It was [also] not just one training, it was several; this was because the facility was working on shifts, so they had to accommodate everyone. During rollout, there was another in-service training just to remind the staff*.

[Nurse 17 – RK]

Moreover, the training was supplemented by regular visits for support and monitoring:

*Then we had regular visits from [the originator], often enough for us to deal with whatever issues we were having on the system.*

[Nurse 2 – MM]

The training was simple and accommodated varying levels of skills:

*Some people could not use computers, but it was made as basic as possible to include those that are not computer literate.*

[Nurse 17 – RK]

However, some users’ competency and reactions to the training were influenced by age:

*For the young ones, it was easy for them. For the old ones, it was a problem because they wanted to understand what they were ticking as they were pressing.*

[Data capturer - 1]

In response, the originators sought assistance from data capturers, acting as an immediate source of help:

*We had person in each clinic who would be there all day long. So for the first day or two they would sit with them [older users] when they completed it, until they became comfortable with it.*

[Originator 1]

#### Support and Feedback Channels

To supplement the training, the originators had different people involved to provide support to end users, including IT technicians:

*We provided desktop support, we were the first line of support. My role firstly was on all applications and IT projects running within the district*.

[IT Technician]

While data capturers were already involved, especially in the early stages, admin people also assisted with various tasks:

*We also had three admin people, one for each clinic that assisted in the clinic to help people with the training, setting up the system and checking that the computers are all working*.

[Originator 1]

The originators also established communication channels to receive feedback from users:

*We had a little book for users to report issues and to add anything, and an E-tick admin person was there. I also got information from data capturers and I would also go and check on everybody and they would give me feedback. We would report back to them monthly, usually*.

[Originator 1]

#### Perceived Benefits of System Use

End users reported several benefits of the system, with some sharing how it improved continuity of care:

*At times you find that the client may have been seen previously but the file could not be found. But if the client’s details were captured on the previous visit, you could see it in the E-tick. So it helped with continuation of care.*

[Nurse 11 – AR]

For other users, such as clerks, this reduced patient waiting times:

*It also reduced the waiting period for the patient, because the first thing that we do is to register the patient and create a folder on the E-tick system. It’s easier to use than retrieving the manual files, searching the files in the cabinets. When you’re using an E-tick, you just search on the system.*

[Admin Clerk 8 – AR]

Facility managers also found operational benefits, such as quicker service delivery:

*The benefits were that the patient consultation times were shortened. It was a little bit faster. That was the best advantage that I’ve seen from the E-tick.*

[Operations Manager 18 – RK]

Some users shared that the system improved data quality and access to it:

*It improved the reporting of data. We had very reliable, consistent, and accurate data, at all times of the day.*

[Administrative officer 19 – RK]

For some, the system enabled the monitoring of the clinic’s performance:

*We are able to evaluate ourselves on the number of patients that we are seeing. When our stats are sent to the DHIS, they can see that we are really performing.*

[Nurse 4 – MM]

#### Perceived Challenges of System Use

Users reported several challenges with using the system, including the simultaneous use of the system with the manual:

*To be brutal, they [users] were against it at first. They did not understand why we’re implementing extra work for them.*

[Administrative officer 19 – RK]

For some participants, the early days before mastering the system were difficult:

*If you’re not used to it yet, it takes time to get used to anything, especially if you’re overloaded with a lot of patients.*

[Nurse 14 – MM]

In some cases, short staffing led nurses to do administrative work, as one nurse shared:

*I think it was mostly shortage of staff, sometimes I had to enter a new patient into the E-tick as a nurse, which would waste time and to delay the queue. You’d end up having to turn some patients back. So it needs us to be well staffed*.

[Nurse 12 – AR]

In response, the originators designated roles for receptionists, clerks, and data capturers as the first point of contact for patient registrations.

There were concerns about the hardware and infrastructure that already existed in the facilities: “The [DOH’s] ICT equipment was very outdated. Some of the computers are so old and they haven’t been updated.” (Originator 1 – R). Some users had challenges with the network infrastructure:

*Our network system was failing us a lot. You’d be prepared to start the day and notice that you have no network*.

[Nurse 12 – AR]

In response to this challenge, a back capture function was introduced, which allowed information to be captured into the system at a later stage. In one site, hardware was frequently stolen:

*First disadvantage was theft. I’d say more than more than 70% of the tablets disappeared. All the rooms that had extension cords, but in less than two weeks, all the rooms had no extension cords. They also stole the chargers.*

[Administrative officer 19 – RK]

In one clinic, the piloting coincided with ongoing protests by nurses:

*When we were trying to start the system, the nurses and others would be outside complaining about the conditions in the clinic [a protest organized by unions]. Most of the nurses were not working. There were other issues in the clinic. I think they saw the E-tick as another thing that was worth protesting about, because it increased their workload.*

[Originator 1 – R]

### Halt and Barriers to Scale Up and Institutionalization: Outer Setting Domain (2021)

#### Changing Developers

Despite its relative success in implementation, several complications affected the continued use and development of the system. One of the complications came when there was a change in the software development company:

*Originally [funders] were working with [a software developing company], who were helping with developing district programs. They were taken over by [another company] so the people that worked on our system were taken off the project, and a new team took over the development*.

[Originator 1 – R]

This affected further developments:

*We needed to update the system to make sure it’s fine. Then it got stuck in that development phase because of the new developers that we have*.

[Originator 1 – R]

Some of the previous work was undone by the new developers:

*There were a lot of upgrades to the system, such as integration with the DHIS. But they removed some of the functionalities that were working well in the process. We were never happy with the final products that we got from them.*

[Originator 1 – R]

#### Funding Cuts

The change of developers brought higher costs:

*They [funders] agreed to pay for the additional development, but the new developers came with a lot of red tape and were quite expensive. The funding got cut and they could only give us a small amount to continue with the development*.

[Originator 1 – R]

Moreover, the funders were eventually withdrawn from operating in the health district:

*They [funders] moved out of the district, because their funding got canceled completely. So what’s happening with their funding will affect our funding*.

[Originator 1 – R]

This had an impact on keeping the system operational:

*So because it’s not open source, it was built on the Microsoft platform and the cost to keep it running is high. It’s also web based so no offline capability, and we need constantly running servers*.

[Originator 2 – T]

Thus, the system needed new funders:

*We’re looking for partners to fund the service, license costs and the implementation in facilities. To date, we haven’t found anyone, so it’s been put to sleep*.

[Originator 2 – T]

#### Provincial and District Support

The originators had buy-in from district officials, who were in support of the system:

*I had meetings with my chief director, and my director, and they are still very keen. The clinics are still very keen… So the buy-in is there, even after two to three years, but the funding is not there*.

[Originator 1 – R]

However, the provincial health office’s priority was to develop a new system, as the department technician explained:

*Currently the department is rolling out an HIS platform where all other applications will be layered under and integrated into*.

[IT Technician]

He further explained how locally developed systems were not as prioritized:

*There were some contrasting and opposing views. The system was started from scratch but the department in most cases doesn’t want that. They want to go on the market and buy something on the shelf. I don’t think approvals were going to be granted*.

[IT Technician]

The participant further mentions how the IT staff are often not seen as essential to the role of delivering care:

*Sometimes different services compete for resources. They will say it’s essential when it’s a clinic, a doctor or medication, and then we [IT personnel] come last*.

[IT Technician]

The originators also expressed their frustration with navigating such terrain:

*So this is where we are stuck now. I think what is important to me is how difficult it is to roll out a system within the Department of Health, in the politics, the corruption and the agendas of people. It makes the story very complicated because we developed it the bottom up, seeing the need, with the buy-in from the district, and from all the role players*.

[Originator 1 – R]

### Broader Context (Outer Setting Domain)

Senior managers and external stakeholders shared insights into the Department of Health’s challenges in implementing EHRs in South Africa. One manager notes how implementation requires a joint and collaborative effort of various stakeholders:

*I think it’s a complex sociotechnical project to do which requires many different role players and expertise and costs, which needs high level support from the National Department of Health which has been lacking.*

[NPO manager]

The provincial health departments hold most of the decision-making power in digital health spending:

*The national office can come up with guidelines and frameworks and ask for reports but the actual spending on health information systems is done at provincial.*

[NPO manager]

In this case, the provincial health department had decided to prioritize an off-the-shelf system:

*There is a provincial initiative and anything new that comes in has to talk to this one, there should be interoperability…I saw it [E-tick], it works. I tried to support it but we didn't get provincial support from the CIO.*

[Provincial manager]

However, NGOs have been supporting and implementing local efforts to build systems (such as E-tick) in health care facilities:

*NGOs are doing better than the districts and the provincial health in terms of implementing efficient systems. Most efficient systems are systems that have been introduced by NGO’s instead of our provincial department.*

[Provincial IT technician]

Some participants raised a concern over political appointments in key decision-making positions:

*I don’t believe that we have the right people in government with the right industry experience. They are just given the title and suddenly, they now have to make decisions. I think it’s very wrong and it sets them up for failure*.

[NGO Technician]

A health department technician added:

*Most of our activities are driven by politicians while our head of department has his own strategies. We get our mandate from politicians*.

[DoH Technician]

As a result, some decisions are made with bias:

*There are bureaucratic officials in Department of Health national, provincial and local. If they do not like you and your project, they will say you have to go through SETA [a parastatal] which is extraordinarily slow, not particularly technically competent, and staggeringly expensive*.

[NPO manager]

Moreover, one academic shared their experience with the Department of Health’s reluctance:

*I think there’s not sufficient trust on the part of Government certainly in my experience. They are not coming to partner with us. In fact, they’re pushing us away. We only get close when we work with NGO’s and so on. The Government definitely keeps us at arms-length.*

[Health Academic]

Corruption and inefficiencies have also created barriers, with the tender system being one of the issues:

*It [corruption] is part of the tender system in South Africa, unfortunately the tender system is open to abuse*.

[Provincial health manager - IT]

Moreover, past corruption scandals have tainted the reputation of the Department of Health:

*The perception is that you can’t trust the National Department any longer to do anything. No one is going to give the National Department a big pot of money to run a tender until it can show that it’s got systems and people in place who can do it without squandering the money*.

[National health manager]

This mistrust has also led to a reluctance to work within the department:

*Now people are reluctant to work for the department for several reasons. They fear being embroiled in controversy and putting their personal integrity at stake*.

[National health manager]

## Discussions

### Principal Findings

The E-tick system, an innovation, was developed to address the issue of poor data quality stemming from errors in data collection, which were common with the use of paper-based registers. The implementation process involved an iterative co-design process with input from all involved individuals, including end users and stakeholders, to ensure users’ needs were met and to streamline the data entry process while supporting scalability and integration with other systems. Despite the system’s successful piloting and user adoption in the inner setting, the outer setting posed challenges in funding, change of developers, and lack of support from the provincial health government, leading to the implementation stalling. Using the CFIR, we explored the interaction of different elements, actors, and stakeholders and how they facilitated or impeded implementation. As such, we organize this discussion using CFIR domains, highlighting both facilitators and barriers.

### Innovation

Poor data quality is a problem that commonly affects routine monitoring of health outcomes [34]. The E-tick system had a clear relative advantage over paper-based registers to improve data quality and reporting, as errors that frequently compromised data quality and routine reporting were minimized, and users perceived the system as more streamlined than paper-based manual registers. At a national scale, this aligns with South Africa’s policies that promote the digitization of record keeping and the establishment of a national EHR [10,35], a crucial component for strategic purchasing and health monitoring. The E-tick’s co-design process enhanced usability and fostered buy-in, which is critical in adoption [36], yet innovation alone was insufficient to ensure sustainability.

### Individuals

At the level of individuals, the originators showed strong commitment by providing vision, technical expertise, and persistence in driving the implementation of the E-tick system. Leadership practices are known to impact staff motivation, especially when they take a participatory approach [37,38]. Their leadership was instrumental in mobilizing resources, persuading managers and funders to support the system’s adoption, acting as champions along with data capturers. Research shows how champions are crucial in promoting technology adoption through building enthusiasm and providing peer support [39]. End users also improved confidence in using the system as a result, especially after undergoing training, which sustained motivation during the pilot. However, while the successful adoption of the E-tick was due to the contribution of many actors, including external stakeholders, individuals in the inner setting were more affected by decisions in the outer setting. This reflects a broader challenge in digital health implementation in South Africa, that for locally developed systems to succeed, appropriate actors must be in place to provide leadership, vision, and sustained support, within an enabling environment that fosters continuity and long-term success [37,40].

### Inner Setting

Within the inner setting, several facilitators supported early implementation, such as the co-design process and extensive user training. The originators successfully engaged a wide range of stakeholders, including senior managers and funders, which was pivotal in overcoming initial financial and political barriers. Involving end users was also crucial in enhancing the system’s usability and promoting buy-in, an important aspect when ensuring effective user adoption [41,42]. Moreover, users’ recognition of the system’s value in improving data quality reinforced their motivation to adopt the system. Studies in other low- to middle-income countries show that organizational commitment is a critical success factor to sustain user motivation during piloting phases [43]. However, the sustained use of the E-tick required stable infrastructure, which often posed challenges for many users in the form of interrupted internet connectivity and power cuts. This mirrors challenges reported in other LMICs, where infrastructural fragilities, such as poor internet connectivity and unstable power supply, are common challenges to EHR adoption [44]. Moreover, without dedicated budgets to overcome these challenges, such as the use of mobile internet devices and backup power solutions, this creates uncertainty about sustained use.

### Implementation Process

The implementation process revealed a mix of strengths and gaps, which were closely linked to the role of individuals and the inner setting. A key strength was the iterative co-design approach, which ensured that the system was not only technically functional but also responsive to user needs and compatible with existing workflows. Participatory design and user engagement are widely recognized in implementation science as best practice, as they enhance user ownership, adaptability, and long-term adoption, especially as most implementation efforts fail due to user resistance [45,46]. Thus, involving both clinicians and managers at early stages of the implementation process helped to align the E-tick system with user realities, reducing resistance to change. However, this process was undermined by a lack of a long-term financing mechanism and failed provincial support, despite the demonstrated proof of concept at the pilot phase. Therefore, while the implementation process was well executed, the system lacked the institutional anchoring and support for long-term use, a critical success factor of digital health efforts [47].

### Outer Setting

We found that the most significant challenges were encountered in the outer setting, particularly the withdrawal of financial support, the change of developers, and the lack of support from the provincial health government due to its lack of capacity. Studies show that one of the main challenges to the initiation of digital health initiatives, including EHRs in South Africa, has been the poor governance and lack of political will [48,49]. For instance, the appointment of people without the required skills and competence has led to a chain of events that affect institutions such as the Gauteng provincial health department [50]. As a result, corruption has eroded the public’s trust in the government. These issues are not isolated but rather reflective of the entrenched political economy dynamics that routinely affect digital health innovations in South Africa. Political appointments, who are put in place because they are easily manipulated, often make decisions driven by political interests rather than professional judgment, leading to the political control of government institutions and their spending [51]. As such, patronage networks often produce politically aligned rather than skilled appointments. This leaves room for corruption, irregular spending, fraud, and poor oversight to mitigate them, with procurement processes being vulnerable to perverse incentives, with contracts awarded to less qualified but politically connected vendors. Although efforts have been made to uncover large-scale corruption in South Africa, the necessary mechanisms have not yet been put in place to deal with the challenges [52,53]. Perverse incentives undermine sustainability as officials face pressure to launch new initiatives instead of sustaining existing ones to ensure full expenditure of budgets.

The administrative fragmentation can be seen as a product of South Africa’s federal system, which means provinces can develop their own health information systems (bottom-up) or acquire them through separate procurement processes (top-down) [8]. In this case, despite the provincial government’s preference for a top-down approach by opting for an off-the-shelf system over the E-tick, they were unable to implement the former. High-income countries such as the United Kingdom, the United States, and Australia have taken top-down, bottom-up, and middle-out approaches, respectively, with varying outcomes [54]. Bottom-up approaches often build on existing systems, such as the E-tick, using interoperability standards to integrate them across facilities. In contrast, top-down approaches replace local systems with a centralized EHR, but this can be too complex, limiting flexibility and local adaptability, as in the case of the United Kingdom [55]. Therefore, our analysis shows that a single centralized model is not necessarily more effective, as lessons from these experiences, including Australia, suggest that a middle-out approach is more effective, as this blends both by offering central guidance while allowing for local systems to gradually align with national plans [56].

Implementing EHRs requires significant investment, vision, robust management systems, and strong oversight to sustain them, making effective leadership essential for success. Strong leadership plays an important role in guiding decision making, mobilizing the right expertise, and establishing mechanisms to prevent corruption, which increases the likelihood of successful implementation, particularly for large-scale EHRs [57]. Middle-income countries like South Africa and China have the capacity to finance and support EHR adoption, but such efforts can be undermined by corruption and poor governance [49,58]. In some cases, poor leadership and governance stand as significant barriers to achieving universal health coverage and strengthening health systems [59].

### Implications for Policy and Practice

Our findings highlight several implications for policy and practice. One of them is the strengthening of accountability in health systems through transparent procurement systems and independent oversight to neutralize the effects of corruption and political patronage. In such cases, procurement frameworks could include pilot-to-scale pathways, where bottom-up innovations such as the E-tick could be formally assessed against national priorities and considered for integration with provincial and national systems. These steps could create a more enabling environment for similar innovations to scale sustainably. Moreover, while the E-tick system was locally hosted, it also incorporated cloud functionality, aligning with the cloud-based EHR architectures promoted in both the NDHS and the national data and cloud policy of South Africa [10,60]. Such policies can be leveraged to mitigate barriers such as reliance on facility servers or hardware failures, challenges already observed with the E-tick, which hold important lessons for similar middle-income country contexts.

Our findings also carry implications for digital health equity, a concept that examines how structural inequities shape the successes and challenges of digital health interventions [61]. In South Africa’s dual health system, where private-sector facilities often have better infrastructure and resources than the public sector, grassroots innovations like E-tick are left to face compounded challenges, especially when implemented in low-resource contexts [62]. The stalled scale-up reflects not only outer system or structural barriers by the government but also the broader inequities in digital infrastructure, institutional support, and resourcing across the country’s health system [63].

### Limitations

Our study incorporates perspectives from a diverse range of stakeholders, including higher-level managers, decision makers, academics, and end users from recipient facilities. This diversity helped in capturing nuanced perspectives from different positions within the system and in closing information gaps through triangulation. A limitation of our study is that data collection from the implementation team, especially clinicians, occurred over a year after the system had halted. As a result, some participants struggled to accurately recall past events, while others had moved to different jobs by the time of data collection. As a result, formal member checking with participants was not possible due to staff turnover and the discontinuation of the E-tick project. However, we validated emerging findings through iterative review with coauthors who included experts in South African health systems and originators who led the implementation from the beginning and had contextual expertise.

### Conclusions

In conclusion, the E-tick system is a valuable innovation that was developed from the ground up with user input. While the system showed promise, its long-term sustainability was undermined by external factors such as funding cuts and poor support, political appointments, and corruption, which led to the unwillingness of provincial officials to support local initiatives such as the E-tick. Such bureaucratic inefficiencies also affected the capacity to move forward with another system. This study demonstrates that without alignment to broader factors such as governance, corruption, and skilled leadership, innovative systems such as the E-tick will struggle to be implemented.

## Supplementary material

10.2196/73831 Multimedia Appendix 1 Summary of findings by implementation stage, alignment with Consolidated Framework for Implementation Research (CFIR) domains, applicable constructs, thematic findings, and illustrative quotes.

## Abbreviations

CFIR: Consolidated Framework for Implementation Research
DHIS: District Health Information Software
E-tick: electronic tick register
EHR: electronic health record
HPRS: Health Patient Registration System
NDHS: National Digital Health Strategy
