# Supplementary material for: Exploring Factors Associated With the Stalled Implementation of a Ground-Up Electronic Health Record System in South Africa: Qualitative Insights From the E-Tick Case Study Using the Consolidated Framework for Implementation Research (CFIR)
Source: JMIR Med Inform. 2026 Jan 12;14:e73831. doi: 10.2196/73831 (PMC12795486; doi:10.2196/73831)
Supplement: Multimedia Appendix 1 [file medinform-v14-e73831-s001.docx]

Table S1. Summary of findings by implementation stage, alignment with Consolidated Framework for Implementation Research (CFIR) domains, applicable constructs, thematic findings, and illustrative quotes.

| Implementation stage and CFIR domains and constructs (applicable) | Thematic findings | Illustrative quotes |
| --- | --- | --- |
| **Origins and purpose** | | |
| Innovation: Relative advantage, Design quality and packaging, and Adaptability | The E-Tick was developed to address poor data quality from paper-based tick registers, offering a clear improvement in accuracy and efficiency. Its adaptable design allowed integration with DHIS[^a^](https://jmir.kriyadocs.com/web_preview?doi=73831&project=medinform&customer=jmir#table4fn1), and it improved upon a basic Excel system. | - *They were trying to lessen discrepancies of data. So primarily, I saw e-tick as that main role. But what was communicated was that it would help us manage a patient and go paperless.* [Nurse 19 – RK] - *That’s how we developed the E-tick. It was basically to collect the data electronically and to be able to feed it directly into the DHIS, in a way that is easy for the nurses in the consultation rooms.* [Originator 1] |
| Individuals: Knowledge and beliefs about intervention and Self-efficacy | Originators believed in the intervention’s value and understood how it could address system inefficiencies. They also felt confident in their ability to deliver it due to prior experience with data systems. | - *[Originator 1] approached us with this idea that to remove the paper registers from facilities to capture the data electronically using mobile devices. So we had a big interest in this, obviously, because it will improve our data.* [Originator 2] |
| Inner setting: Tension for change | There was shared recognition of the need to improve data quality, which created urgency among district-level leaders and implementers. | - *We wanted to lessen discrepancies of data, in the sense that when in the consultation rooms, we've got this tick register book, that needs to be calculated by the clinician, the clinician ticks and then calculate at the end, sometimes with errors*. [Nurse 17 – RK] |
| Outer setting: Patient needs and resources and External policies and incentives | Persistent audit failures and national reporting needs created external pressures to improve data systems. However, national policy did not yet support bottom-up digital solutions like E-tick. | - *The problem in South Africa is that the people who decide where the funding goes don’t take data management records management seriously. They want this wealth of data so they put in these big paper registers, but they don’t look at the process it takes to get that data.* [Originator 2] |
| **Initial development** | | |
| Individuals: Individual identification with the organization and Self-efficacy | The two originators took ownership of the system, identifying strongly with the project’s goals and acting as champions to drive development and gain buy-in. | - *[Originator 1] was very good on the clinician side and I was very good on the software side. I had been working with my team [funders] for many years before we started the E-tick, and I developed many systems with them.* [Originator 2] |
| Inner setting: Network and communication, Compatibility, and Available resources | Effective collaboration between clinicians, developers, and funders enabled iterative co-design. The system was built to align with existing clinic workflows and facility infrastructure, although resource gaps were bridged by external funders. | - *My role was to check infrastructure, to see if it’s there. But they also installed some private infrastructure because of some policies with our network which would require a lot of approvals before we can run it.* [IT Technician] - *One person came to train us and it was a teaching. So whoever was in the room was taught, then we continued practicing on our own and helping and assisting each other when we struggle.* [Nurse 3 – MM] |
| Outer setting: Cosmopolitanism | Relationships with external partners (NGOs, funders, developers) were central to initial system development and support. | - *We approached [funders] for assistance and presented our idea to them…as they were our long term funding partner and they were also very involved in data collection…so at that point they agreed then we had quite a lot of meetings.* [Originator 1] - *In a normal software development cycle, you scope your business requirements, hand it over to the team, and they will go develop what they think you want and then return to show it to you. We took a different approach. We were involved in the development, sitting with them [developers] every step of the way instead of waiting for weeks for changes. This worked extremely well*. [Originator 2] |
| Implementation process: Planning and Engaging | The system was introduced through structured and consultative meetings and workshops with facility managers and end users. Clear planning with stakeholder involvement promoted ownership. | - *We were called to a district meeting, and this was pitched, the intention was discussed with us...So the facilities that were going to be pilots were selected.* [Assistant Director 17 – RK] - *They called us for like a mini meeting to inform us about E-tick and told us that we were one of the only clinics currently using it, and they were piloting to see if it’s going to work.* [Nurse 10 – AR] |
| **Piloting and expansion** | | |
| Individuals: Self-efficacy and Knowledge and beliefs | Staff competency varied, especially across age groups as younger staff adapted quickly while older staff needed more support. Nonetheless, most users believed in the system’s usefulness after onboarding. | - *For the young ones, it was easy for them. For the old ones, it was a problem because they wanted to understand what they were ticking as they were pressing.* [Data capturer - 1] - *At times you find that the client may have been seen previously but the file could not be found. But if the client’s details were captured on the previous visit, you could see it in the e-tick. So it helped with continuation of care.* [Nurse 11 – AR] |
| Inner setting: Readiness for implementation and Structural characteristics | Facilities had computers, although some infrastructure upgrades (eg, hardware, cabling) were needed. Clinics varied in preparedness, but funder support helped bridge gaps. | - *They [funders] assisted us and made sure that each room had a network cable. Sometimes there were no plugs, so they put in the plugs. They put in the routers.* [Originator 1 – R)] - *Our network system was failing us a lot. You’d be prepared to start the day and notice that you have no network.* [Nurse 12 – AR] - *First disadvantage was theft. I’d say more than 70% of the tablets disappeared. They were secured in those stands that use the unique screws to lock them after but people took them out without even needing to remove the screws*. [Nurse 19 – RK] |
| Implementation process: Executing, Reflecting and evaluating, and Engaging | Continuous user training, feedback loops, and system adaptations occurred during this stage. System benefits (faster service, better reporting) were acknowledged by users. | - *There were different training categories, because the roles were different like, clerks, clinicians and so on. It was [also] not just one training, it was several; this was because the facility was working on shifts, so they had to accommodate everyone. During rollout, there was another in-service training just to remind the staff.* [Nurse 17 – RK] - *It also reduced the waiting period for the patient, because the first thing that we do is to register the patient and create a folder on the E-tick system. It’s easier to use than retrieving the manual files.* [Admin Clerk 8 – AR] |
| **Halt (Stall)** | | |
| Individuals: Champion and Other personal attributes | The originators remained committed but were unable to maintain momentum due to systemic barriers beyond their control. | - *The Provincial office decided to develop a whole new system, ignoring all the existing systems that were there. When I asked them, they said, obviously they want to keep using the existing systems and we need to have a bilateral agreements on how to keep the e-tick information into the system*. [Originator 1 – R] |
| Inner setting: Available resources and Implementation climate | Funding cuts and change of developers led to diminished support for sustained adoption. Facility staff were frustrated, and momentum was lost despite initial enthusiasm. | - *Eventually the funders moved out of [the district] and basically all the funding was cut finally, and they’ve given the system over to us [originators] to manage. So they are not part of the funding anymore but would only fund the basics like holding the data up to when we get a new funder.* [Originator 1] |
| Outer setting: External policy and incentives, Peer pressure, and Leadership engagement | Provincial authorities did not support E-tick due to political interference, lack of support and intersectoral collaboration, corruption in the tender system, and distrust in government procurement. | - *There is a provincial initiative and anything new that comes in has to talk to this one, there should be interoperability…I saw it [e-tick], it works. I tried to support it but we didn't get provincial support from the CIO*. [Provincial manager] - *I don’t believe that we have the right people in government with the right industry experience…They are just given the title and suddenly, they now have to make decisions. I think it’s very wrong and it sets them up for failure.* [NGO Technician] |

^a^DHIS: District Health Information Software.
